# Supplementary material for: High SLC2A1 expression associated with suppressing CD8 T cells and B cells promoted cancer survival in gastric cancer
Source: PLoS One. 2021 Mar 18;16(3):e0245075. doi: 10.1371/journal.pone.0245075 (PMC7971512; doi:10.1371/journal.pone.0245075)
Supplement: S1 File — (PDF) [file pone.0245075.s001.pdf]

# High SLC2A1 expression associated with suppressing CD8 T cells and B cells promoted cancer survival in gastric cancer.

Kyueng-Whan Min[1*], Dong-Hoon Kim[2*], Byoung Kwan Son[3], Kyoung Min Moon[4], So Myoung Kim[5], Md. Intazur Rahaman[5], So Won Kim[5], Eun-Kyung Kim[6], Mi Jung Kwon[7], Young Wha Koh[8], Il Hwan Oh[3]

[1]Department of Pathology, Hanyang University Guri Hospital, Hanyang University College of Medicine, Guri, Gyeonggi-do, Republic of Korea

[2]Department of Pathology, Kangbuk Samsung Hospital, Sungkyunkwan University School of Medicine, Seoul, Republic of Korea

[3]Department of Internal Medicine, Eulji Hospital, Eulji University School of Medicine, Seoul, Republic of Korea

[4]Department of Internal Medicine, Gangneung Asan Hospital, University of Ulsan College of Medicine, Gangneung, Gangwon-do, Republic of Korea

[5]Department of Pharmacology, University of Ulsan College of Medicine, Asan Medical Center, Seoul, Korea

[6]Department of Pathology, Eulji Hospital, Eulji University School of Medicine, Seoul, Republic of Korea

[7]Department of Pathology, Hallym University Sacred Heart Hospital, Hallym University College of Medicine, Anyang, Gyeonggi-do, Republic of Korea

[8]Department of Pathology, Ajou University School of Medicine, Suwon, Gyeonggi-do, Republic of Korea

**Running title:** SLC2A1 in gastric cancer

**Corresponding Author:** Byoung Kwan Son,

Department of Internal Medicine, Eulji Hospital, Eulji University School of Medicine, 68 Hangeulbiseok-ro, Nowon-gu, Seoul, Republic of Korea [01830]

Tel: +82-2-970-8209, Fax: +82-2-970-8621, E-mail: sbk1026@eulji.ac.kr

*These authors contributed equally to this work.

# Supplementary Information

### Reverse transcription polymerase chain reaction (RT-PCR)

MKN-45 cells (1 x $10^6$) were seeded into 60-mm dishes and incubated at 37°C and 5% CO2 for 24 hours. After one day, the cells were treated with DMSO or 1 µM retinoic acid for 24 hours. RNAisoPLUS (9109, TAKARA, Japan) was used to isolate RNA following the manufacturer's instructions. For cDNA synthesis, PrimeScript™ RT Master Mix (RR036A, TAKARA, Japan) was used following the manufacturer's instructions. RT-PCR was performed using Solg™ 2X EF-Taq PCR Smart mix 2 (SEF02-M50h, Solgent, Korea). The cycle was repeated 20 times for GAPDH and 22 times for GLUT1 for 40 seconds at 58°C for annealing and 30 seconds at 72°C for polymerization. The size of the PCR product was 273 bp (GAPDH) and 220 bp (GLUT1). The nucleotide sequences of the primers were as follows: GAPDH forward primer 5'-CTCATGACCACAGTCCATGCCATC-3' and reverse primer 5'-CTGCTTCACCACCTTCTTGATGTC-3'; GLUT1 forward primer 5'-ACTCCATCATGGGCAACAAG-3' and reverse primer 5'-TGCCGACTCTCTTCCTTCAT-3. Molecular biology grade agarose (11AGAH0500, MPbio, USA), TAE (T2002, bioWORLD, Korea) and GelGreen Nucleic Acid Gel Stain (41005, Biotium, USA) were used to make a 1% agarose gel, and PCR products were run on Mupid-exU (AD140E, TAKARA, Japan). The results were analyzed with an iBright CL1000 (Thermo Fisher, USA).

### Immunoblotting

MKN-45 cells (1 x $10^6$) were seeded on 60-mm dishes. After 24 hours, retinoic acid (1 µM) or DMSO was added and incubated at 37°C in a 5% CO2 incubator for 24 hours. After cell harvest, a mammalian protein prep kit (37901, QIAGEN, Germany) was used for protein extraction. The concentration of proteins was measured using protein assay reagents A, B and S (5000113~5, BIO-RAD, USA). Protein samples were loaded into EzWay PAG Precast Gels, 4-12% (KG5012, KOMABIOTECH, KOREA) with Tricine Running Buffer (KTR030, KOMABIOTECH, KOREA) and GangNam-STAIN™ Prestained Protein Ladder (24052, iNtRON, KOREA). Proteins were transferred from the gel to a PVDF membrane (1620177, BIO-RAD), and Tris-Glycine Transfer Buffer (K0341001, KOMABIOTECH, KOREA) was used. Membranes were blocked with 5% skim milk (232100,

BD Diagnostic Systems, UK) in 1% TBST at room temperature for 1 hour. Then, membranes were incubated with monoclonal anti-β-actin (A5441, Sigma-Aldrich, USA) and anti-glucose transporter GLUT1 antibody (ab40084, Abcam, UK) diluted in General-BLOCK Solution (TLP-115.1G, Translab, Korea) at 4°C overnight. The washing process with TBST was carried out 3 times for 10 min each. Goat anti-mouse IgG (1706516, Bio-Rad) in General-BLOCK Solution was added to the membranes at room temperature for 1 hour. The washing process with TBST was again carried out 3 times for 10 min. Membranes were treated with the West-Q Chemiluminescent Substrate Plus Kit (W3651-012, GenDEPOT, USA), and the protein blots were analyzed by iBright CL1000.
