# Supplementary material for: High SLC2A1 expression associated with suppressing CD8 T cells and B cells promoted cancer survival in gastric cancer
Source: PLoS One. 2021 Mar 18;16(3):e0245075. doi: 10.1371/journal.pone.0245075 (PMC7971512; doi:10.1371/journal.pone.0245075)
Supplement: S1 Fig — The GLUT1 protein has a size of 55 kDa but varies slightly in shape depending on cell, antibody or experimental conditions. (PDF) [file pone.0245075.s002.pdf]

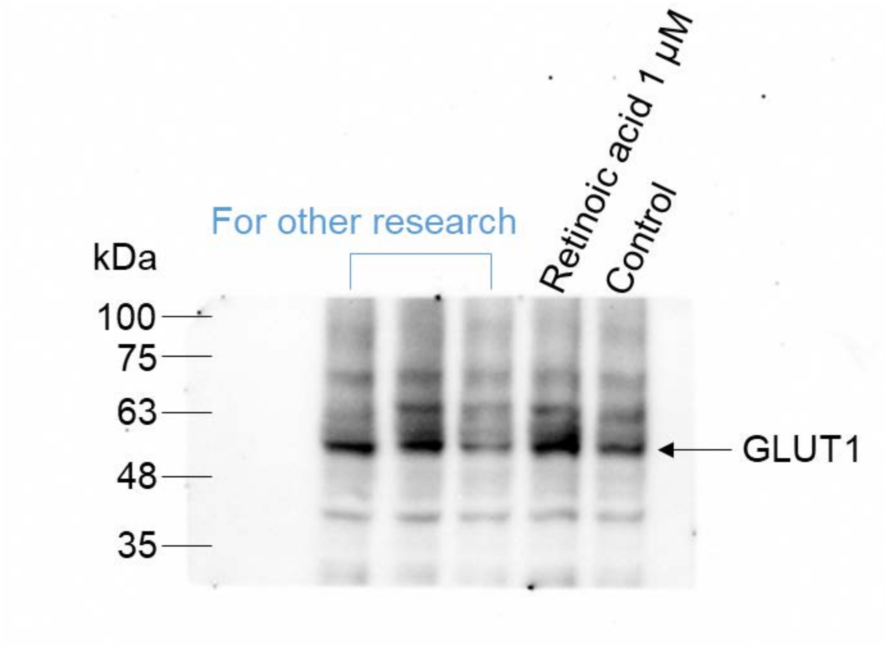

S1 Fig. Full-length gels and blots**.** The GLUT1 protein has a size of 55 kDa but varies slightly in shape depending on cell, antibody or experimental conditions.
