# Supplementary material for: High SLC2A1 expression associated with suppressing CD8 T cells and B cells promoted cancer survival in gastric cancer
Source: PLoS One. 2021 Mar 18;16(3):e0245075. doi: 10.1371/journal.pone.0245075 (PMC7971512; doi:10.1371/journal.pone.0245075)
Supplement: S1 Table — (PDF) [file pone.0245075.s003.pdf]

S1 Table Clinicopathological parameters of Eulji cohort

No	GLUT	Age (yo)	T stage	N stage	Location	Size (cm)	Lauren classification	Histological grade	LI	VI	PNI	EBV
1	-	>65	3	0	Cardia, fundus or body	>3	intestinal	moderately	+	+	+	-
2	-	>65	2	0	Cardia, fundus or body	>3	intestinal	well	+	-	-	-
3	-	>65	1	0	Cardia, fundus or body	≤3	diffuse	signet	-	-	-	-
4	-	>65	1	0	Cardia, fundus or body	>3	intestinal	moderately	+	-	-	-
5	+	>65	3	0	Cardia, fundus or body	>3	intestinal	moderately	+	-	+	-
6	+	>65	3	3	Antrum or pylorus	>3	mixed	moderately	+	+	+	-
7	+	>65	3	1	Antrum or pylorus	>3	mixed	poorly	+	+	+	-
8	+	>65	1	0	Antrum or pylorus	>3	intestinal	well	-	-	-	-
9	+	>65	2	0	Cardia, fundus or body	>3	mixed	poorly	+	+	+	-
10	+	>65	1	0	Antrum or pylorus	≤3	intestinal	well	-	-	-	-
11	+	>65	1	0	Antrum or pylorus	≤3	intestinal	well	-	-	-	-
12	-	≤65	1	0	Cardia, fundus or body	≤3	intestinal	moderately	-	-	-	-
13	-	>65	1	0	Antrum or pylorus	≤3	mixed	poorly	-	-	-	+
14	+	>65	3	0	Cardia, fundus or body	≤3	diffuse	signet	-	-	-	-
15	+	≤65	1	1	Cardia, fundus or body	≤3	intestinal	moderately	+	-	-	-
16	+	>65	1	0	Cardia, fundus or body	≤3	intestinal	moderately	-	-	-	-
17	-	≤65	3	0	Antrum or pylorus	≤3	mixed	poorly	-	-	+	-
18	+	>65	2	1	Cardia, fundus or body	>3	intestinal	moderately	+	+	-	-
19	-	≤65	3	0	Antrum or pylorus	≤3	mixed	poorly	-	-	+	-
20	+	≤65	3	2	Cardia, fundus or body	>3	mixed	poorly	+	+	-	-
21	+	>65	1	0	Antrum or pylorus	≤3	intestinal	well	-	-	-	-

22	-	>65	3	3	Cardia, fundus or body	>3	diffuse	signet	+	+	-	+
23	-	>65	2	3	Cardia, fundus or body	>3	diffuse	signet	+	+	-	+
24	-	>65	1	0	Antrum or pylorus	≤3	intestinal	well	-	-	-	-
25	-	>65	1	0	Antrum or pylorus	>3	intestinal	moderately	-	-	-	-
26	+	≤65	2	3	Cardia, fundus or body	>3	diffuse	poorly	+	+	-	-
27	-	>65	4	3	Cardia, fundus or body	>3	diffuse	poorly	-	-	+	-
28	+	≤65	3	2	Antrum or pylorus	>3	mixed	poorly	+	+	-	-
29	-	≤65	4	3	Cardia, fundus or body	>3	diffuse	signet	+	-	+	-
30	+	≤65	1	0	Antrum or pylorus	≤3	intestinal	moderately	-	-	-	-
31	-	≤65	1	0	Cardia, fundus or body	≤3	diffuse	signet	-	-	-	-
32	+	≤65	4	0	Cardia, fundus or body	>3	mixed	poorly	-	-	+	-
33	-	≤65	1	0	Cardia, fundus or body	≤3	intestinal	well	-	-	-	+
34	-	≤65	1	0	Antrum or pylorus	≤3	mixed	poorly	-	-	-	-
35	+	≤65	3	0	Cardia, fundus or body	>3	mixed	poorly	+	+	+	-
36	+	>65	1	0	Cardia, fundus or body	≤3	intestinal	moderately	-	-	-	-
37	+	≤65	3	3	Antrum or pylorus	>3	intestinal	moderately	-	-	-	-
38	+	≤65	4	3	Antrum or pylorus	>3	diffuse	signet	+	+	+	-
39	+	>65	3	3	Antrum or pylorus	>3	intestinal	moderately	+	-	+	-
40	-	>65	1	0	Cardia, fundus or body	≤3	intestinal	well	-	-	-	-
41	-	>65	3	0	Antrum or pylorus	≤3	diffuse	signet	-	-	+	-
42	+	>65	1	0	Antrum or pylorus	>3	mixed	poorly	-	-	-	-
43	+	≤65	3	0	Antrum or pylorus	>3	mixed	signet	-	-	+	-
44	+	>65	1	1	Antrum or pylorus	≤3	intestinal	moderately	+	-	-	-
45	+	>65	3	0	Antrum or pylorus	>3	intestinal	moderately	-	-	-	-

46	-	>65	1	0	Cardia, fundus or body	>3	diffuse	signet	-	-	-	-
47	+	≤65	4	2	Antrum or pylorus	>3	mixed	poorly	+	-	+	-
48	-	≤65	4	0	Antrum or pylorus	≤3	diffuse	signet	-	-	-	-
49	+	>65	3	1	Cardia, fundus or body	>3	intestinal	moderately	+	-	+	-
50	+	>65	1	2	Antrum or pylorus	>3	intestinal	moderately	+	-	-	-
51	+	>65	3	3	Antrum or pylorus	>3	mixed	poorly	+	+	-	-
52	-	≤65	1	0	Antrum or pylorus	≤3	diffuse	signet	-	-	-	-
53	+	>65	3	0	Antrum or pylorus	>3	intestinal	moderately	+	+	+	-
54	+	≤65	1	2	Antrum or pylorus	>3	mixed	signet	+	-	-	-
55	-	>65	1	0	Cardia, fundus or body	≤3	intestinal	well	-	-	-	-
56	-	≤65	2	0	Antrum or pylorus	>3	mixed	poorly	-	-	-	-
57	-	>65	2	1	Cardia, fundus or body	>3	diffuse	signet	+	-	-	-
58	+	>65	3	0	Cardia, fundus or body	>3	diffuse	signet	-	-	-	-
59	-	>65	1	0	Antrum or pylorus	>3	intestinal	moderately	+	-	-	-
60	-	>65	4	3	Antrum or pylorus	>3	diffuse	signet	+	+	+	+
61	-	>65	1	0	Antrum or pylorus	>3	intestinal	well	+	-	-	-
62	+	>65	4	3	Antrum or pylorus	>3	diffuse	signet	+	-	+	-
63	-	>65	1	0	Cardia, fundus or body	≤3	diffuse	signet	-	-	-	-
64	+	>65	1	3	Cardia, fundus or body	>3	mixed	poorly	+	+	+	-
65	-	>65	1	0	Antrum or pylorus	>3	mixed	signet	-	-	-	-
66	+	>65	4	0	Antrum or pylorus	>3	intestinal	signet	+	+	-	-
67	-	>65	3	1	Antrum or pylorus	>3	intestinal	moderately	+	+	+	-
68	-	≤65	3	3	Antrum or pylorus	>3	diffuse	poorly	+	-	+	-
69	-	≤65	1	0	Cardia, fundus or body	>3	intestinal	moderately	-	-	-	-

70	+	≤65	1	0	Antrum or pylorus	≤3	intestinal	moderately	-	-	-	-
71	+	>65	3	0	Antrum or pylorus	>3	intestinal	moderately	+	-	-	-
72	-	≤65	4	2	Cardia, fundus or body	>3	intestinal	moderately	+	-	+	-
73	-	>65	1	0	Cardia, fundus or body	>3	intestinal	moderately	-	-	-	-
74	+	≤65	1	0	Antrum or pylorus	>3	mixed	signet	+	+	-	-
75	+	>65	2	0	Cardia, fundus or body	≤3	intestinal	moderately	+	-	-	-
76	-	>65	2	1	Cardia, fundus or body	>3	intestinal	moderately	+	-	-	-
77	+	>65	3	3	Cardia, fundus or body	>3	intestinal	moderately	+	+	-	-
78	+	>65	1	0	Cardia, fundus or body	≤3	intestinal	moderately	-	-	-	-
79	+	>65	4	3	Antrum or pylorus	>3	mixed	poorly	+	+	-	-
80	+	>65	1	0	Cardia, fundus or body	>3	intestinal	moderately	-	-	-	-
81	-	>65	1	0	Cardia, fundus or body	≤3	diffuse	poorly	-	-	+	-
82	+	>65	4	2	Antrum or pylorus	>3	mixed	poorly	+	-	-	-
83	+	≤65	3	2	Antrum or pylorus	>3	intestinal	moderately	+	-	-	-
84	+	>65	1	0	Antrum or pylorus	>3	intestinal	well	-	-	-	-
85	+	≤65	1	0	Antrum or pylorus	>3	intestinal	well	+	-	-	-
86	-	>65	1	0	Cardia, fundus or body	>3	mixed	poorly	+	-	-	+
87	-	>65	1	0	Antrum or pylorus	≤3	mixed	poorly	-	-	-	-
88	+	>65	1	0	Cardia, fundus or body	≤3	intestinal	moderately	+	-	-	-
89	+	≤65	4	3	Antrum or pylorus	>3	diffuse	signet	+	-	+	-
90	+	>65	1	0	Antrum or pylorus	≤3	intestinal	moderately	-	-	-	-
91	-	>65	3	0	Cardia, fundus or body	≤3	mixed	poorly	-	-	+	-
92	+	>65	3	1	Antrum or pylorus	>3	intestinal	moderately	+	+	+	-
93	+	≤65	1	0	Antrum or pylorus	>3	mixed	signet	-	-	-	-

94	+	≤65	1	0	Antrum or pylorus	>3	intestinal	moderately	-	-	-	-
95	-	>65	1	0	Antrum or pylorus	≤3	intestinal	moderately	-	-	-	-
96	-	>65	1	0	Cardia, fundus or body	≤3	intestinal	moderately	-	-	-	-
97	-	>65	1	0	Cardia, fundus or body	≤3	diffuse	signet	-	-	-	-
98	-	>65	1	0	Cardia, fundus or body	≤3	intestinal	well	-	-	-	-
99	+	≤65	1	0	Cardia, fundus or body	>3	intestinal	well	+	+	-	-
100	-	≤65	1	0	Antrum or pylorus	≤3	intestinal	moderately	-	-	-	-
101	-	≤65	3	0	Antrum or pylorus	>3	diffuse	signet	+	-	-	-
102	+	>65	1	0	Antrum or pylorus	≤3	intestinal	moderately	-	-	-	-
103	-	>65	1	0	Antrum or pylorus	≤3	intestinal	moderately	-	-	-	-
104	-	>65	2	3	Antrum or pylorus	>3	intestinal	signet	+	-	-	-
105	+	>65	1	0	Antrum or pylorus	>3	diffuse	poorly	+	-	-	-
106	-	>65	1	0	Antrum or pylorus	≤3	intestinal	well	-	-	-	-
107	+	>65	3	0	Antrum or pylorus	>3	intestinal	moderately	+	-	-	-
108	-	≤65	1	0	Cardia, fundus or body	>3	intestinal	moderately	-	-	-	-
109	+	>65	1	0	Cardia, fundus or body	≤3	intestinal	moderately	+	-	-	-
110	-	>65	1	2	Cardia, fundus or body	>3	mixed	poorly	-	-	-	-
111	-	>65	1	1	Antrum or pylorus	>3	intestinal	poorly	+	-	-	-
112	-	>65	1	0	Antrum or pylorus	>3	intestinal	well	-	-	-	-
113	+	>65	1	2	Cardia, fundus or body	≤3	intestinal	moderately	+	-	-	-
114	+	≤65	1	0	Antrum or pylorus	≤3	intestinal	moderately	-	-	-	+
115	-	>65	1	0	Antrum or pylorus	≤3	diffuse	signet	-	-	-	-
116	-	>65	1	0	Antrum or pylorus	>3	diffuse	signet	-	-	-	-
117	+	>65	3	3	Antrum or pylorus	>3	intestinal	moderately	+	+	-	-

118	-	≤65	1	0	Antrum or pylorus	≤3	intestinal	moderately	-	-	-	-
119	-	>65	1	0	Antrum or pylorus	>3	mixed	poorly	+	-	-	-
120	-	≤65	1	1	Cardia, fundus or body	>3	diffuse	signet	+	-	-	-
121	-	>65	1	0	Antrum or pylorus	>3	intestinal	moderately	-	-	-	-
122	-	>65	1	0	Antrum or pylorus	≤3	intestinal	well	-	-	-	-
123	-	>65	1	0	Antrum or pylorus	≤3	intestinal	well	+	-	-	-
124	-	≤65	1	0	Cardia, fundus or body	>3	diffuse	signet	-	-	-	-
125	-	>65	1	0	Cardia, fundus or body	≤3	intestinal	poorly	+	-	-	-
126	-	>65	1	0	Antrum or pylorus	≤3	mixed	poorly	-	-	-	-
127	-	≤65	1	0	Antrum or pylorus	≤3	intestinal	moderately	-	-	-	-
128	-	>65	1	0	Antrum or pylorus	>3	mixed	poorly	-	-	-	-
129	-	>65	1	0	Antrum or pylorus	>3	diffuse	poorly	-	-	-	-
130	+	>65	1	1	Antrum or pylorus	≤3	mixed	poorly	-	-	-	-
131	-	>65	1	0	Antrum or pylorus	≤3	intestinal	moderately	-	-	-	-
132	+	≤65	1	0	Antrum or pylorus	≤3	intestinal	moderately	-	-	-	-
133	+	>65	1	0	Antrum or pylorus	≤3	mixed	poorly	-	-	+	-
134	+	>65	1	0	Antrum or pylorus	>3	intestinal	moderately	+	+	-	-
135	+	≤65	4	3	Antrum or pylorus	>3	intestinal	moderately	+	-	+	+
136	-	>65	3	3	Cardia, fundus or body	≤3	diffuse	signet	+	-	+	-
137	-	≤65	3	0	Cardia, fundus or body	≤3	intestinal	moderately	+	-	-	-
138	-	>65	1	0	Cardia, fundus or body	>3	diffuse	signet	-	-	-	-
139	+	≤65	1	0	Antrum or pylorus	≤3	intestinal	well	-	-	-	-
140	+	≤65	4	3	Cardia, fundus or body	>3	diffuse	signet	+	-	+	-
141	+	>65	2	1	Antrum or pylorus	>3	intestinal	moderately	+	-	-	+

142	-	≤65	1	0	Antrum or pylorus	>3	intestinal	well	-	-	-	-
143	+	≤65	1	0	Antrum or pylorus	≤3	intestinal	moderately	-	-	-	-
144	-	>65	4	3	Antrum or pylorus	>3	diffuse	signet	+	-	+	-
145	+	>65	1	0	Cardia, fundus or body	≤3	mixed	poorly	-	-	-	-
146	-	>65	1	0	Antrum or pylorus	≤3	intestinal	well	-	-	-	-
147	-	>65	1	0	Antrum or pylorus	≤3	intestinal	well	-	-	-	-
148	+	>65	1	0	Antrum or pylorus	≤3	intestinal	moderately	-	-	-	-
149	+	>65	1	0	Cardia, fundus or body	≤3	intestinal	moderately	-	-	-	-
150	-	≤65	1	0	Cardia, fundus or body	≤3	diffuse	signet	-	-	-	-
151	-	≤65	1	0	Antrum or pylorus	>3	intestinal	signet	-	-	-	-
152	+	≤65	1	0	Antrum or pylorus	≤3	intestinal	moderately	-	-	-	-
153	-	>65	1	0	Antrum or pylorus	>3	intestinal	well	-	-	-	-
154	-	>65	1	0	Cardia, fundus or body	>3	diffuse	signet	-	-	-	-
155	+	>65	4	3	Antrum or pylorus	>3	mixed	poorly	+	-	+	+
156	+	≤65	1	0	Antrum or pylorus	≤3	intestinal	well	-	-	-	-
157	+	>65	1	0	Antrum or pylorus	≤3	intestinal	moderately	-	-	-	-
158	-	>65	1	0	Cardia, fundus or body	≤3	intestinal	well	-	-	-	-
159	-	>65	1	0	Antrum or pylorus	≤3	intestinal	moderately	-	-	-	-
160	+	>65	3	2	Antrum or pylorus	>3	intestinal	moderately	+	-	+	-
161	-	≤65	3	0	Cardia, fundus or body	>3	diffuse	signet	+	-	+	-
162	+	>65	4	3	Antrum or pylorus	>3	intestinal	moderately	+	+	-	-
163	-	>65	1	0	Antrum or pylorus	≤3	intestinal	moderately	-	-	-	-
164	-	≤65	1	0	Antrum or pylorus	≤3	intestinal	well	-	-	-	-
165	+	≤65	3	3	Antrum or pylorus	>3	intestinal	moderately	+	-	+	-

166	-	>65	3	1	Antrum or pylorus	≤3	mixed	poorly	+	-	-	-
167	+	≤65	1	0	Antrum or pylorus	≤3	intestinal	well	-	-	-	-
168	-	>65	1	0	Antrum or pylorus	≤3	intestinal	well	-	-	-	-
169	-	>65	1	0	Antrum or pylorus	≤3	intestinal	moderately	-	-	-	-
170	-	≤65	1	0	Antrum or pylorus	≤3	intestinal	moderately	-	-	-	-
171	-	>65	1	0	Cardia, fundus or body	≤3	intestinal	well	-	-	-	+
172	-	>65	1	0	Cardia, fundus or body	≤3	intestinal	moderately	-	-	-	-
173	+	>65	1	0	Cardia, fundus or body	≤3	intestinal	well	-	-	-	-
174	+	>65	1	0	Antrum or pylorus	≤3	intestinal	moderately	-	-	-	-
175	+	>65	4	3	Cardia, fundus or body	>3	intestinal	moderately	+	-	-	+
176	-	≤65	4	3	Antrum or pylorus	>3	diffuse	signet	+	-	-	-
177	-	>65	3	3	Antrum or pylorus	>3	diffuse	signet	+	-	+	-
178	+	>65	1	0	Antrum or pylorus	>3	intestinal	moderately	-	-	-	-
179	-	≤65	3	0	Antrum or pylorus	>3	diffuse	signet	-	-	+	-
180	+	>65	4	0	Antrum or pylorus	>3	diffuse	signet	+	-	+	+
181	-	>65	1	0	Antrum or pylorus	>3	intestinal	moderately	-	-	-	-
182	-	>65	1	0	Antrum or pylorus	≤3	intestinal	well	+	-	-	+
183	-	>65	1	0	Antrum or pylorus	≤3	intestinal	well	-	-	-	-
184	+	≤65	1	0	Cardia, fundus or body	≤3	intestinal	moderately	-	-	-	-
185	+	>65	1	0	Antrum or pylorus	≤3	intestinal	moderately	-	-	-	-
186	+	>65	1	0	Cardia, fundus or body	>3	diffuse	signet	-	-	-	-
187	+	>65	3	2	Cardia, fundus or body	>3	intestinal	moderately	+	-	-	-
188	+	>65	3	2	Antrum or pylorus	>3	mixed	poorly	+	-	+	-
189	+	>65	1	0	Cardia, fundus or body	>3	intestinal	moderately	-	-	-	-

190	+	>65	1	0	Antrum or pylorus	≤3	intestinal	moderately	-	-	-	-
191	+	>65	2	3	Cardia, fundus or body	>3	intestinal	moderately	+	-	-	-
192	+	>65	1	0	Cardia, fundus or body	≤3	intestinal	moderately	-	-	-	-
193	-	≤65	1	0	Antrum or pylorus	≤3	intestinal	well	-	-	-	-
194	+	>65	4	3	Cardia, fundus or body	>3	diffuse	poorly	+	+	+	+
195	-	>65	3	1	Antrum or pylorus	>3	mixed	poorly	-	-	+	-
196	+	>65	3	0	Antrum or pylorus	>3	intestinal	moderately	-	+	-	+
197	-	≤65	2	0	Antrum or pylorus	≤3	diffuse	signet	-	-	+	-
198	+	>65	4	2	Antrum or pylorus	≤3	intestinal	moderately	+	-	+	+
199	+	>65	1	0	Antrum or pylorus	>3	intestinal	moderately	-	-	-	-
200	-	≤65	1	0	Antrum or pylorus	>3	intestinal	moderately	-	-	-	-
201	-	≤65	4	1	Cardia, fundus or body	≤3	diffuse	signet	+	-	-	-
202	-	>65	4	2	Antrum or pylorus	>3	mixed	poorly	+	-	+	-
203	+	≤65	1	0	Antrum or pylorus	>3	intestinal	moderately	-	-	-	-
204	-	>65	1	0	Antrum or pylorus	≤3	intestinal	signet	-	-	-	-
205	-	>65	1	0	Antrum or pylorus	≤3	intestinal	signet	-	-	-	-
206	-	≤65	4	3	Cardia, fundus or body	>3	diffuse	signet	+	+	+	-
207	+	>65	1	0	Antrum or pylorus	≤3	intestinal	well	-	-	-	-
208	-	>65	1	0	Antrum or pylorus	>3	intestinal	moderately	-	-	-	-
209	-	>65	4	3	Antrum or pylorus	>3	mixed	signet	+	-	-	-
210	+	>65	1	0	Antrum or pylorus	≤3	intestinal	moderately	-	-	-	-
211	+	≤65	3	3	Cardia, fundus or body	>3	diffuse	poorly	+	-	+	-
212	+	>65	4	3	Antrum or pylorus	>3	intestinal	moderately	+	+	+	-
213	-	>65	1	1	Antrum or pylorus	≤3	intestinal	moderately	+	-	-	-

214	+	>65	2	2	Antrum or pylorus	>3	intestinal	moderately	+	-	-	-
215	+	>65	4	1	Cardia, fundus or body	>3	mixed	poorly	+	+	+	-
216	+	>65	4	2	Cardia, fundus or body	>3	intestinal	moderately	+	-	-	+
217	+	>65	2	0	Cardia, fundus or body	>3	intestinal	well	-	-	-	-
218	+	>65	4	2	Antrum or pylorus	>3	intestinal	moderately	+	-	-	-
219	+	≤65	4	3	Cardia, fundus or body	>3	intestinal	moderately	+	+	+	-
220	+	>65	1	0	Antrum or pylorus	>3	intestinal	signet	-	-	-	-
221	+	>65	3	3	Antrum or pylorus	≤3	intestinal	moderately	+	-	+	-
222	-	≤65	4	1	Cardia, fundus or body	>3	diffuse	signet	+	-	-	-
223	+	≤65	1	0	Antrum or pylorus	≤3	intestinal	well	-	-	-	-
224	-	>65	1	1	Antrum or pylorus	≤3	diffuse	signet	-	-	-	-
225	-	>65	3	0	Antrum or pylorus	≤3	intestinal	poorly	+	+	-	-
226	+	≤65	3	3	Antrum or pylorus	≤3	diffuse	signet	+	-	-	+
227	+	>65	2	0	Antrum or pylorus	>3	intestinal	moderately	-	-	-	-
228	-	>65	1	0	Cardia, fundus or body	≤3	intestinal	moderately	-	-	-	-
229	+	>65	4	3	Antrum or pylorus	>3	mixed	poorly	+	+	+	-
230	+	>65	4	3	Antrum or pylorus	>3	intestinal	moderately	+	+	-	-
231	+	>65	2	0	Antrum or pylorus	>3	intestinal	moderately	-	-	-	-
232	+	>65	3	3	Antrum or pylorus	>3	intestinal	moderately	+	-	-	-
233	-	≤65	2	3	Antrum or pylorus	>3	intestinal	poorly	+	-	-	-
234	-	≤65	1	0	Cardia, fundus or body	≤3	diffuse	signet	-	-	-	-
235	+	>65	1	0	Antrum or pylorus	≤3	intestinal	well	-	-	-	-
236	+	>65	4	3	Cardia, fundus or body	>3	mixed	signet	+	-	-	-
237	-	≤65	1	0	Antrum or pylorus	>3	diffuse	signet	-	-	-	-

238	+	>65	2	1	Antrum or pylorus	>3	intestinal	moderately	+	+	-	-
239	-	≤65	1	0	Antrum or pylorus	≤3	diffuse	signet	-	-	-	-
240	+	>65	4	3	Cardia, fundus or body	>3	mixed	signet	+	+	+	+
241	+	>65	4	3	Antrum or pylorus	>3	diffuse	signet	+	+	-	-
242	-	≤65	1	0	Antrum or pylorus	>3	intestinal	well	-	-	-	-
243	-	>65	1	0	Cardia, fundus or body	>3	diffuse	signet	-	-	-	-
244	-	>65	1	0	Antrum or pylorus	>3	intestinal	moderately	-	-	-	-
245	-	>65	2	3	Antrum or pylorus	≤3	intestinal	poorly	-	-	+	-
246	+	>65	1	0	Antrum or pylorus	>3	intestinal	moderately	-	-	-	-
247	-	>65	1	0	Cardia, fundus or body	>3	intestinal	poorly	-	-	-	-
248	+	≤65	1	0	Antrum or pylorus	≤3	intestinal	poorly	+	+	-	+
249	+	>65	1	0	Antrum or pylorus	≤3	intestinal	poorly	-	-	-	-
250	-	>65	1	0	Cardia, fundus or body	>3	intestinal	moderately	-	-	-	+
251	+	≤65	1	1	Antrum or pylorus	≤3	intestinal	moderately	-	-	-	+
252	+	>65	1	0	Antrum or pylorus	≤3	intestinal	well	-	-	-	-
253	+	>65	1	0	Antrum or pylorus	>3	intestinal	moderately	-	-	-	+
254	+	>65	1	0	Antrum or pylorus	>3	intestinal	well	-	-	-	-
255	+	>65	3	1	Cardia, fundus or body	≤3	intestinal	poorly	+	-	-	+
256	-	>65	1	0	Antrum or pylorus	≤3	diffuse	poorly	-	-	-	-
257	+	>65	4	3	Antrum or pylorus	>3	intestinal	moderately	+	+	-	+
258	+	>65	4	2	Antrum or pylorus	≤3	intestinal	moderately	-	-	-	+
259	-	>65	4	3	Cardia, fundus or body	≤3	intestinal	moderately	+	-	-	+
260	+	>65	4	3	Cardia, fundus or body	>3	intestinal	poorly	+	+	-	+
261	-	>65	4	1	Antrum or pylorus	>3	intestinal	signet	-	-	-	-

262	-	>65	1	0	Antrum or pylorus	≤3	intestinal	moderately	+	-	-	-
263	+	≤65	1	0	Cardia, fundus or body	>3	intestinal	moderately	-	-	-	-
264	+	>65	4	3	Cardia, fundus or body	≤3	intestinal	moderately	+	+	-	+
265	-	>65	1	0	Cardia, fundus or body	>3	intestinal	moderately	-	-	-	-
266	+	>65	3	3	Antrum or pylorus	>3	intestinal	moderately	+	+	-	-
267	-	>65	3	3	Cardia, fundus or body	>3	diffuse	signet	+	+	-	+
268	+	>65	4	2	Antrum or pylorus	>3	intestinal	moderately	+	-	+	-
269	-	≤65	1	0	Cardia, fundus or body	≤3	diffuse	signet	-	-	-	-
270	+	>65	3	1	Cardia, fundus or body	≤3	intestinal	moderately	+	+	-	+
271	-	≤65	1	0	Cardia, fundus or body	≤3	diffuse	signet	-	-	-	+
272	-	>65	1	0	Antrum or pylorus	≤3	intestinal	well	-	-	-	+
273	-	≤65	2	2	Cardia, fundus or body	≤3	diffuse	signet	+	-	-	-
274	+	>65	3	3	Antrum or pylorus	>3	intestinal	poorly	+	+	-	-
275	+	>65	4	3	Antrum or pylorus	>3	diffuse	signet	+	-	-	+
276	-	≤65	1	0	Antrum or pylorus	≤3	intestinal	moderately	-	-	-	+
277	+	>65	4	3	Cardia, fundus or body	>3	intestinal	moderately	+	-	-	+
278	-	≤65	1	0	Cardia, fundus or body	≤3	diffuse	signet	-	-	-	-
279	-	>65	4	3	Antrum or pylorus	≤3	intestinal	moderately	+	-	-	-

LI, lymphatic invasion; VI, vascular invasion; PNI, perineural invasion;, EBV; Epstein-Barr virus
